# Rational Doping Strategy to Build the First Solution‐Processed p‐n Homojunction Architecture toward Silicon Quantum Dot Photodetectors

**DOI:** 10.1002/smsc.202400367

**Published:** 2024-10-06

**Authors:** Batu Ghosh, Hiroyuki Yamada, Kazuhiro Nemoto, Wipakorn Jevasuwan, Naoki Fukata, Hon‐Tao Sun, Naoto Shirahata

**Affiliations:** ^1^ Research Center for Materials Nanoarchitectonics (MANA) National Institute for Materials Science (NIMS) 1‐1 Namiki Tsukuba 305‐0044 Japan; ^2^ Department of Physics Triveni Devi Bhalotia College Raniganj West Bengal 713347 India; ^3^ Graduate School of Chemical Sciences and Engineering Hokkaido University Kita 13, Nishi 8, Kita‐ku Sapporo 060‐8628 Japan

**Keywords:** electric impurity doping, p‐n homojunction, photodiodes, silicon quantum dots, solution‐processed optoelectronics

## Abstract

Semiconductor p‐n homojunction is a requisite building block of operating transistors and diodes which make up the modern electronic circuits and optoelectronic applications. However, it has been so far limited to bulk form of single crystals such as silicon (Si) or gallium arsenide. Herein, a brand‐new method of constructing p‐n homojunction architectures that breaks through the limitation is presented. Colloidal inks of p‐type and n‐type Si quantum dots (QDs) are synthesized by thermal disproportionation of (HSiO_1.5_)_*n*_ doped with boron or phosphorus, followed by surface ligand engineering. Analysis combining UV photoelectron spectroscopy, electron spin resonance, and current–voltage characteristics confirms that an orthogonal solvent trick makes clean interfaces between n‐type and p‐type SiQD layers without disruption on film formation. The forward and reverse current–voltage characteristics of the diode, along with various spectroscopic characterizations, demonstrate the formation of the first p‐n homojunction of SiQDs. The self‐powered photodiode provides a tunable response specific to the wavelength.

## Graphical abstract

This article reports a novel solution‐processed method of constructing p‐n homojunction architectures using p‐type and n‐type Si QDs that are dispersed in two orthogonal solvents. The p‐n homojunction demonstrates typical *I*–*V* characteristics which clearly show diode‐like rectification and it is used further as a photodiode to detect light efficiently even at zero bias voltage conditions.

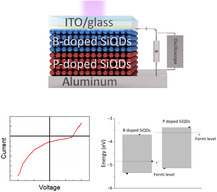

## 1 Introduction

Electronic impurity doping in a single crystalline semiconductor has led to dramatic advances in the control of electronic and optoelectronic properties, enabling most of the functionalities that enrich our social life today and human civilization, from transistors to a variety of optoelectronic devices, including solar cells and laser diodes. The p‐n junction diode that is constructed at homo‐ or heterojunction interface between p‐type and n‐type semiconductors is the heart of device architecture to realize those functionalities.

Colloidal quantum dots (CQDs) have attracted increasing attention as promising photoactive materials for next‐generation optoelectronics due to their unique characteristics including size‐dependent spectral tunability, multiexciton generation, and bandgap engineering.^[^ ^1^, ^2^, ^3^, ^4^, ^5^, ^6^ ^]^ Furthermore, they offer added benefits of scalable synthesis and solution processability compatible with printable technology, thus allowing for large‐area manufacturing, including roll‐to‐roll processes and low‐cost fabrication without using vacuum apparatus. As with bulk crystals, in thin films based on CQDs, electronic impurity doping allows controlling the concentration and mobility of charge carriers to build a p‐n junction. Doping in CQDs, however, differs significantly from typical processes used in bulk crystal. A couple of methods have been reported for compound semiconductor CQDs, such as surface doping, substitution of impurity element at a lattice site, and introduction of impurity element into an interstitial site. For surface functionalization, since their energy structures can be tuned by variation of surface ligand chemistry,^[^ ^7^, ^8^, ^9^ ^]^ the difference in surface ligand allows for having the dexterity to vary doping‐type (i.e., p‐type and n‐type characteristics). Initial attempts have been subjected to PbS; so far, 1,2‐ethanedithiol or carboxylate are representative capping ligands for p‐type conductivity,^[^ ^10^, ^11^ ^]^ while n‐type conductivity is given by substituting the divalent sulfur ions for monovalent halogen ions.^[^ ^12^, ^13^ ^]^ Incorporating heterovalent impurities that can be substituted for cations introduces additional carriers in CQDs. In chalcogenide CQDs such as PbS and CdSe, the divalent cations are replaced by Ag^+^ ions to provide positive free carriers for p‐type conductivity.^[^ ^14^, ^15^ ^]^ InAs CQD is a typical n‐type,^[^ ^16^ ^]^ whereas incorporation of substitutional doping with hetero‐valent Zn^2+^ ion to replace In^3+^ provides free holes for p‐type character.^[^ ^17^ ^]^ In spite of those great efforts, little progress has been made on homogeneous p‐n junctions in CQDs and their use for optoelectronic applications with acceptable performances.

Silicon (Si), a second abundant element in the earth's crust, is a typical indirect bandgap semiconductor. A wafer of Si is a material cornerstone for microelectronics and solar cells for more than half of century. Electronic impurity doping in a single crystalline bulk Si is a well‐established technique; phosphorus (P) and boron (B) are doped to form n‐type and p‐type semiconductors, respectively. These crystals benefit from controlling carrier concentration and mobility through impurity doping, as it allows building p‐n junctions as a key to operating optoelectronic devices in the modern industry. The electronic dopants are also being used in other forms of Si such as nanowires to form p‐n homojunction and heterojunction for device applications including solar cell and phototransistor.^[^ ^18^, ^19^ ^]^ Considering the technological applications of unique properties emerged by the quantum confinement effects such as multiexciton generation, a more intriguing challenge would be especially to dope impurity in Si QDs. To date, many efforts have been attempted to reveal the correlation between optical and structural properties of QDs embedded in bulky oxide or nitride matrixes (e.g., the exact location of dopant atoms), with varying dopant concentration, most notably using boron and phosphorus.^[^ ^20^, ^21^, ^22^, ^23^, ^24^ ^]^ Such bulky materials containing doped QDs have been used to fabricate heterojunction optoelectronic devices such as solar cells,^[^ ^25^, ^26^, ^27^ ^]^ but there is little chance of creating a p‐n homojunction as long as the QDs are embedded. Impurity doping to self‐standing QDs has been achieved by liberating the doped QDs from the SiO_*x*_ matrix with hydrofluoric (HF) acid etching.^[^ ^28^, ^29^, ^30^, ^31^, ^32^ ^]^ Determination of the Fermi level, which is subjected to hydrogen‐terminated QDs doped with boron or phosphorus, was first reported in 2015 using Kelvin probe microscopy.^[^ ^33^ ^]^ In the following year, photoelectron yield spectroscopic (PYS) study reported that Fermi level, highest‐occupied molecular orbital (HOMO), and lowest‐unoccupied molecular orbital (LUMO) energies of QDs codoped with boron and phosphorus are dependent on their diameters.^[^ ^34^ ^]^ In principle, it should also be possible to provide extra carriers to Si CQD via electric impurity doping to build a p‐n homojunction, but this has not yet been realized. In this work, we report, for the first time, emergent p‐n homojunction where p‐type and n‐type self‐standing Si QD layers have overlapped each other. The fabrication of photodiode devices using CQDs, which efficiently detect irradiated UV light with zero bias, opens up great possibilities for a new paradigm in Si optoelectronics.

## 2 Results and Discussion

### 2.1 Synthesis of Colloidal p‐Type and n‐Type Si QDs

**Scheme 1:**
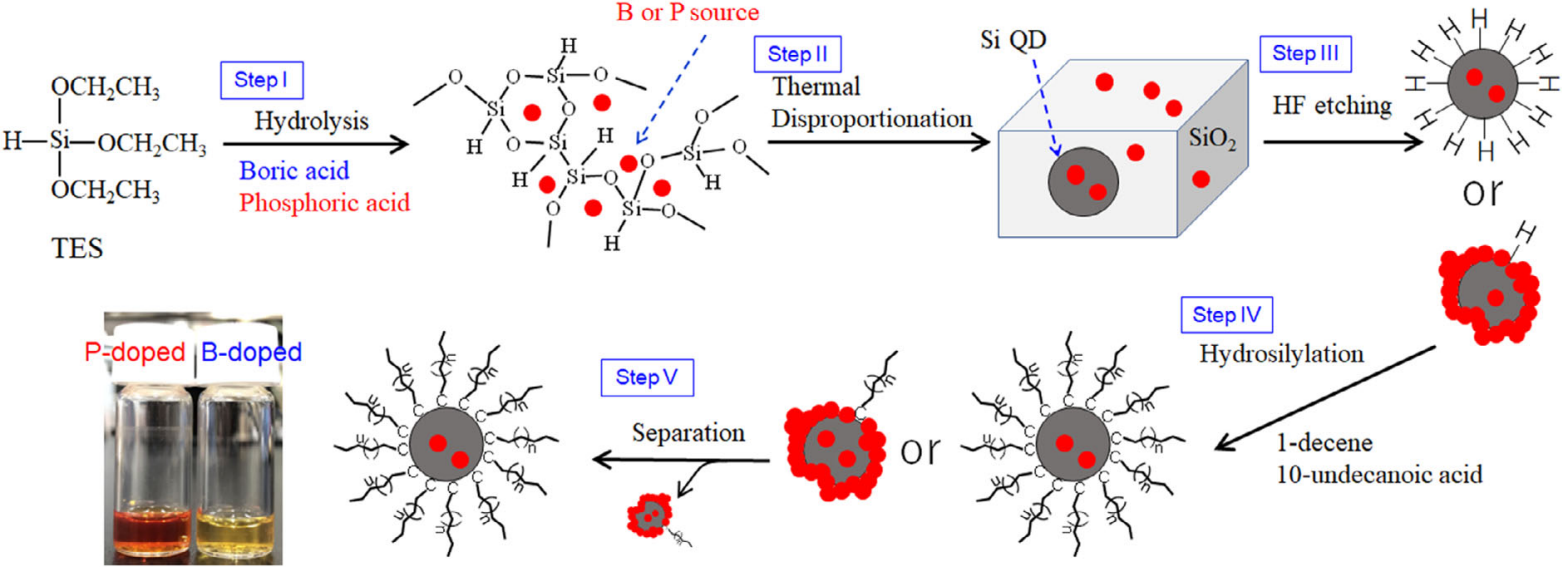
Synthesis of colloidal inks of phosphorus‐doped and boron‐doped Si QDs dispersed in ethanol and toluene, respectively.

B‐doped and P‐doped Si QDs were synthesized according to **Scheme** **1**. Triethoxysilane (TES) was hydrolyzed in the presence of boric acid or phosphoric acid (step I), followed by filtered, washed, and dried. It has been known that the hydrolysis product of TES corresponds to amorphous hydrogen silsesquioxane, that is, (HSiO_1.5_)_*n*_,^[^ ^35^ ^]^ which can be disproportionated to Si and SiO_2_ at temperatures above 1000 °C.^[^ ^36^ ^]^ In this work, the hydrolysis product was heated at 1100 °C (step II). The resulting powder was uniformly dark brown in color. Then, the powder was treated with HF acid to liberate the hydrogen‐terminated Si QDs from SiO_2_ matrix (step III), followed by thermal hydrosilylation of 1‐decene or 10‐unidecenoic acid (step IV). As the reaction proceeds, the outermost Si atoms were terminated by monolayers of decane or undecanoic acid. It has been known that reaction solution turns colored transparent liquid when monomolecular coverage exceeded ≈16%,^[^ ^37^, ^38^ ^]^ because those monolayers inhibit aggregation of QDs to result in complete isolation of the QDs in the good solvent. In the present study, however, the reaction solution remained slightly cloudy even after a long reaction time for hydrosilylation. This suggests that impurity ions attached to Si atoms in the outermost layer of QDs inhibited hydrosilylation, resulting in lower monolayer coverage for poor solution solubility in some of the QDs. In step V, the QDs with low monolayer coverages were removed by centrifugation to give colored transparent solutions of B‐doped and P‐doped QDs (see digital photographs in Scheme 1).

**Figure 1:**
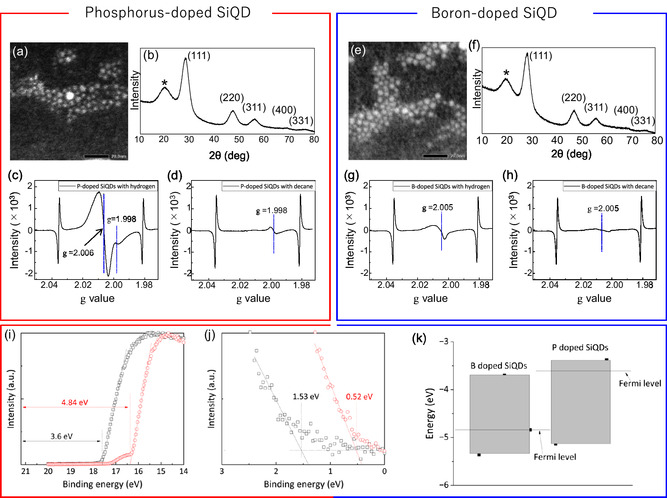
Summary of physical and electronic properties of P‐doped and B‐doped Si QDs. a,e) HR‐TEM images, b,f) XRD patterns, d,h) ESR spectra, and i,j) UPS spectra of the P‐doped and B‐doped Si QDs terminated with decane and undecanoic acid monolayers, respectively. c,g) ESR spectra of hydrogen‐terminated Si QDs served as standards. k) Possible energy structures of the doped Si QDs terminated with surface monolayers. The XRD peaks marked with an asterisk are attributed to organic ligands.^[^ ^54^ ^]^

Scanning transmission electron microscopic (STEM) images shown in **Figure** **1** exhibit round‐shaped nanoparticles with average diameters of 2.1 and 2.5 nm prepared using (a) phosphoric acid and (e) boric acid. Observation of nanoparticles isolated and dispersed without aggregation indicated the termination of their surfaces with organic monolayers of longer chain length,^[^ ^39^ ^]^ such as decane or 10‐undecanoic acid. X‐ray powder diffraction (XRD) patterns shown in Figure 1b,f confirmed that the drop‐cast nanoparticles consist of a face‐centered diamond cubic crystal structure of Si, and the diffraction angles were almost identical to those of bulky Si. Chemical bonding states of the hydrogen‐terminated samples were characterized by X‐ray photoelectron microscopy (XPS) as shown in Figure S1 (Supporting Information). As expected, XPS Si2p spectrum was split into two peaks centered at 99.3 eV for Si^0^ and 103.6 eV for Si^4+^ due to slight oxidation of Si even after HF treatment.^[^ ^38^ ^]^ It is known that the metallic boron and boron oxide exhibit spectral peaks at 188 and 193 eV, respectively.^[^ ^40^ ^]^ Observation of a broad peak centered at 188.5 eV in the XPS B1s spectrum indicated that the majority is B^0^ while B_2_O_3_ is absent. The doped boron atoms exhibited weak Raman peaks at 620 cm^−1^ for ^11^B and 644 cm^−1^ for ^10^B (see Figure S2, Supporting Information), consistent with the previous work.^[^ ^40^ ^]^ XPS P2*p* spectrum exhibited two peaks centered at 129.6 and 134.4 eV. The prominent peak was attributed to the P^0^ bonding state and the broad tail to suboxides. These signals observed in the XPS B1s and P2p spectra disappeared after the purification of the hydrosilylated samples via step V shown in Scheme 1, suggesting that the signals observed as peaks have originated from the impurity atoms deposited on sample surfaces. Unlike bulk Si crystal giving TO phonon line at 521 cm^−1^ in a symmetric vibration region, the momentum is not necessarily conserved in a size range smaller than the Bohr radius (≈5 nm).^[^ ^41^ ^]^ The relaxation of the momentum selection rule allows the occurrence of Raman‐active modes away from the Brillouin zone center, resulting in the peak broadening asymmetrically and redshifting as shown in Figure S2, Supporting Information. Such a size‐dependent characteristic could be observed in optical properties. Optical absorbance and photoluminescence (PL) spectra of the hydrosilylated samples are shown in Figure S3 (see Supporting Information). There was no significant difference in terms of absorbance between the B‐doped and the P‐doped samples. PL spectra of the P‐doped and B‐doped samples were centered at 1.77 and 1.66 eV, consistent with the relationship between size and bandgap for the undoped SiQDs.^[^ ^41^, ^42^ ^]^ In addition, we measured the PL spectra by varying excitation wavelengths between 300 and 400 nm for each doped QD sample. The results are shown in Figure S3, Supporting Information. Obviously, the PL peak position remains unchanged with varying excitation wavelengths, indicating that the emission is not originating from surface trap states or other defect‐related trap states. PL quantum yields (QYs) of both hydrogen‐terminated samples were smaller than 2% but enhanced to 41% and 21% after hydrosilylation of 1‐decene and 10‐undecenoic acid, respectively.

Electron spin resonance (ESR) measurements were performed at 4.2 K using an X‐band ESR spectrometer with a magnetic field modulation of 100 kHz to discuss the possible doping sites in the crystalline structure of Si QDs terminated with undecanoic acid and decane monolayers (see Figure 1d,h) while ESR spectra of the hydrogen‐terminated samples were used as standard (see Figure 1c,g). In Figure 1c, the ESR signal was clearly observed at *g* = 1.998, corresponding to that of conduction electrons in P‐doped crystalline Si,^[^ ^28^ ^]^ indicating that phosphorus atoms are doped in substitutional sites of the crystalline Si core. As expected, this signal remained after hydrosilylation of 1‐decene. This was evidence of successful n‐type doping although XPS P2*p* spectrum did not give any peak. On the other hand, we see the appearance of signals at *g* = 2.006, possibly due to the presence of dangling bonds as a defect formed in an amorphous structure.^[^ ^43^, ^44^ ^]^ This is consistent with a previous study that reported that the outermost layer of hydrogen‐terminated Si QD consists of an amorphous structure with many dangling bonds as nonradiative channels.^[^ ^45^ ^]^ Interestingly, the defect‐derived signals disappeared after hydrosilylation of 1‐decene, suggesting the disappearance of amorphized surface Si layer. This observation is consistent with our previous study that has reported that the passivation with decane monolayers suppresses surface reconstruction to preserve the diamond cubic lattice (i.e., inhibits amorphization of QD surface) in a broad range from the center toward the near surface in Si QD.^[^ ^46^ ^]^ It can be discussed that the absence of dangling bonds as nonradiative channels provoked enhanced PLQY to 41%. On the other hand, in the case of B‐doped Si QDs capped with hydrogen atoms, the conduction electron signal at *g* = 1.998 was not observed. The result is quite reasonable because conduction electrons do not exist in p‐type B‐doped Si QDs. The hole‐related ESR signals in Si are difficult to observe and are not observed under normal conditions. A defect‐related signal was observed at around *g* = 2.005. The shape of the signal is asymmetric, suggesting that there are at least two types of defects with similar structures. Although the *g*‐value is slightly different from that in the case of P‐doped Si QDs, it can be discussed that the defects are dangling bond‐type defects which exist in the surface amorphous layer of the QDs as in the case of P‐doped Si QDs. It is noted that the signal at *g* = 2.005 was significantly weakened by hydrosilylation of 10‐undecenoic acid, consistent with the improved PLQY due to a decrease of nonradiative channels. Unlike P dopant that prefers to take the *sp*3 configuration, B impurities tend to be more stable even near the Si surface.^[^ ^47^ ^]^ Therefore, the fact that a small peak remains at *g* = 2.005 even after the hydrosilylation suggests that the B dopants are responsible for supplying holes as electronic charge carriers in the outermost layer of the QDs.

Energy structures of the hydrosilylated QDs were explored through ultraviolet photoelectron microscopy (UPS) as depicted in Figure 1i,j. This investigation delved into the intriguing effects of doping on these nanostructures. Using the UPS spectra, the work function, representing the energy required to remove an electron from the material's surface, was determined to be −3.6 eV for P‐doped QDs and −4.84 eV for B‐doped QDs, both relative to the vacuum level. The rise in photoelectron intensity nearest the Fermi energy (*E*_F_) is usually assigned as the valence band maximum energy (*E*_VBM_). To extract the *E*_VBM_ with respect to *E*_F_ (*E*_F_−*E*_VBM_), the rise in photoelectron intensity is fitted to a line and extrapolated to a point that intersects a linear fit to the baseline. The UPS measurement revealed that the VBM energy for the B‐doped QDs was ≈−5.36 eV, while for P‐doped QDs, it was ≈−5.13 eV with respect to vacuum. On the other hand, the bandgap of 1.66 and 1.75 eV for the B‐doped and P‐doped were calculated from the PL peak photon energy, respectively. Thus, the conduction band minima (CBM) were calculated to be −3.7 and −3.37 eV respectively for the B‐doped and P‐doped QDs. Fermi‐level positions were indicative of semiconductor behavior. For the B‐doped QDs, it lay closer to the valence band, confirming p‐type behavior, while for the P‐doped QDs, it was closer to the conduction band, indicating n‐type behavior. Based on the UPS study, the positions of the valence band, conduction band, and Fermi level are illustrated in Figure 1k, offering a visual insight into the intricate energy landscape of the p‐type and n‐type doped Si‐QD system. Rectangular blocks have been used to show schematically the bandgap with energy scale in the vertical axis. The top line of the blocks represents the VBM position whereas the bottom line of the blocks represents the CBM position. The horizontal black line drawn between band gaps represents the Fermi level, which separates the occupied and unoccupied states within the QD. This visual representation offers a clear understanding of the energy structure and the positioning of key energy levels within the QDs.

### 2.2 Fabrication of p‐n Homojunction Photodiode

**Figure 2:**
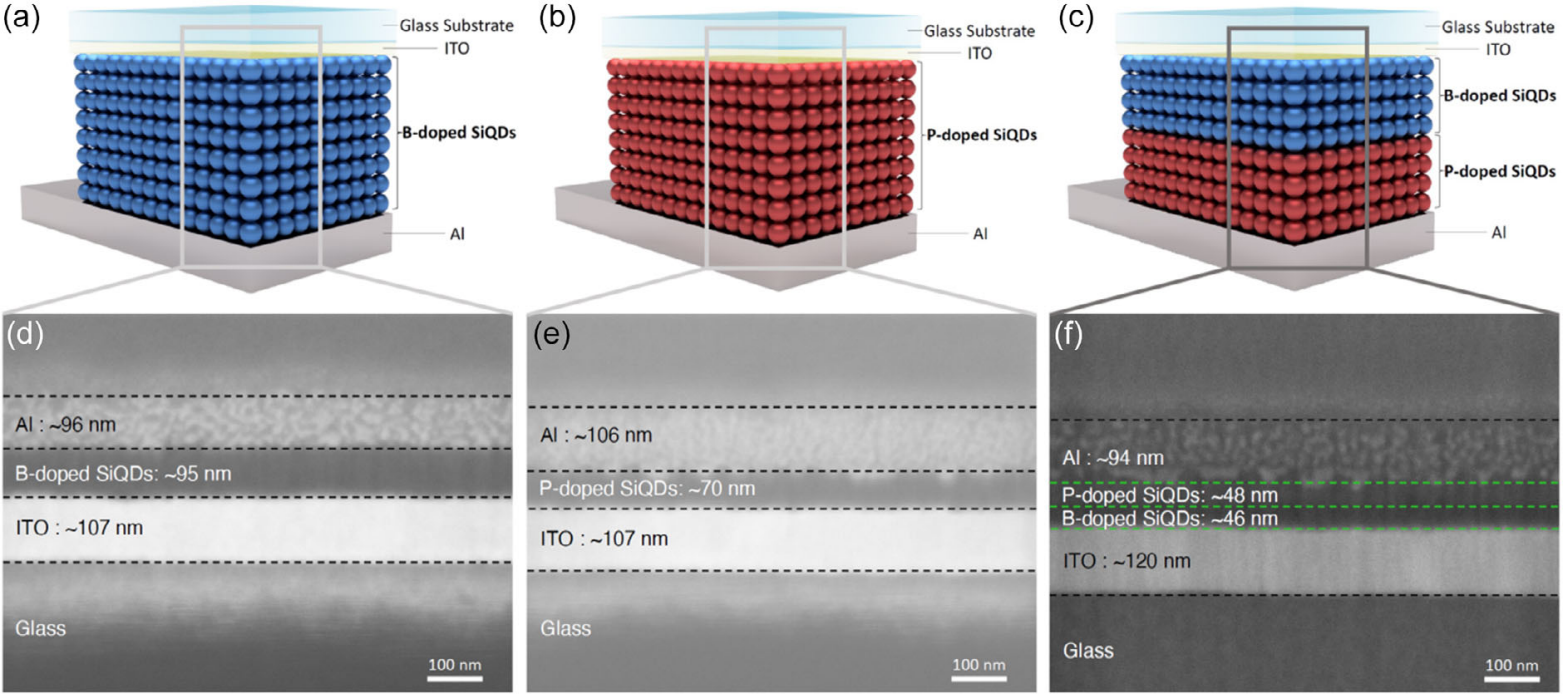
Schematic illustrations and cross‐sectional SEM images of a) p‐type, b) n‐type, and c) p‐n junction photodetectors. Real cross sectional SEM images of d) p‐type, e) n‐type, and f) p‐n junction photodetectors.

**Figure 3:**
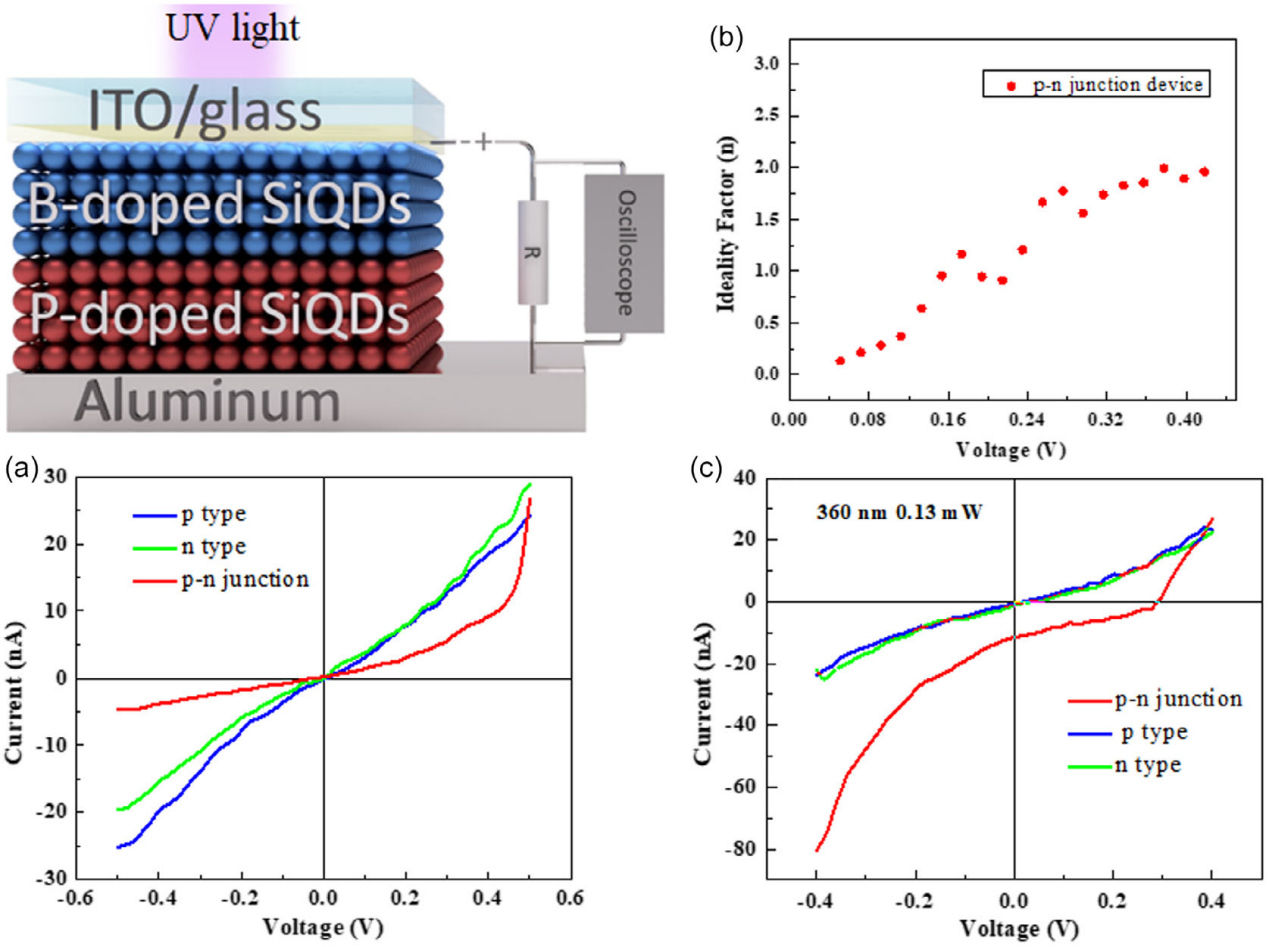
a) Dark IV characteristics of only p‐type, only n‐type, and p‐n junction device. b) Ideality factor of the p‐n junction diode. c) *I*–*V* characteristics of three devices under light of 360 nm with optical power 0.13 mW.

One of the challenges in allowing to build p‐n homojunction through the solution process is how to stack p‐type QD layers on top of n‐type QD layers, and vice versa, without mixing. In this work, we used two different ligands (i.e., decane and undecanoic acid) for n‐type and p‐type Si QDs which make them soluble in two orthogonal solvents (i.e., toluene and ethanol). **Figure** **2**a,b are cross‐sectional SEM images of devices with p‐type or n‐type Si QD layers sandwiched between ITO (as a cathode) and Al (as an anode) electrodes, respectively. The QD layers were spin‐coated onto the surface of glass substrates covered with thin film of ITO, after which an Al thin film of ≈100 nm was deposited. The thicknesses of the QD layers were tuned to ≈95 and ≈70 nm and these devices were used as a reference. To prepare the device shown in Figure 2c, p‐type QDs were spin coated on the ITO surface, followed by n‐type QDs. The thickness of each QD layer was adjusted to half the thickness of the QD layers used for p‐type only and n‐type only devices to compare the optoelectronic performance between the three devices. The dark *I*–*V* characteristics shown in **Figure** **3**a confirm that for the p‐type only and the n‐type only devices, *I*–*V* curves were linear in behavior which are obvious for any semiconductor and that the conductivities are nearly same or comparable. In contrast, the *I*–*V* characteristics of the p‐type/n‐type stacked device show a clear rectification behavior, suggesting that the depletion layer formed at the interface between the p‐type and n‐type layers gives the diode characteristics. Therefore, current conduction was favorable under forward bias, whereas current conduction was low under reverse bias conditions. The *I*–*V* curve was fitted with the diode equation^[^ ^48^ ^]^

(1) n(V)=qkT[dV/d(lnI)]

where *n*(*V*) is ideality factor, *q* is charge, *k* is boltzman constant, and *T* is temperature.

Figure 3b shows ideality factors plotted as a function of bias voltage. Ideality factor increases as forward bias voltage increases in the region where the effect of the series resistance is small and then increases slowly with increasing forward bias where the effect of the series resistance comes into play in the *I*–*V* characteristics. This indicates the appearance of p‐n junction behavior.^[^ ^48^, ^49^, ^50^ ^]^ Furthermore, we studied the photoresponses of the three devices upon UV light irradiation (*λ* = 360 ± 5 nm, 0.13 mW) using a xenon lamp, and the results are shown in Figure 3c. Increasing trends of photocurrent for the p‐type only and n‐type only devices are quite similar. Due to the difference in magnitude of work function between the cathode and the anode, a little photovoltaic property has been aroused. Open‐circuit voltage (*V*_oc_) and short circuit current (*I*_sc_) of p‐type only device was 0.44 V and 1.7 nA, respectively while n‐type only device produced 0.49 V of *V*_oc_ and 1.65 nA of *I*_sc_. In the p‐n homojunction device, where the rectifying behavior of the diode was evident from the *I*–*V* characteristics, the values of *V*_oc_ and *I*_sc_ were improved to 0.65 V and 15.5 nA. Such an enhanced photovoltaic performance would be evidence of the formation of p‐n homojunction at the interface between the B‐doped and P‐doped Si QD layers.

**Figure 4:**
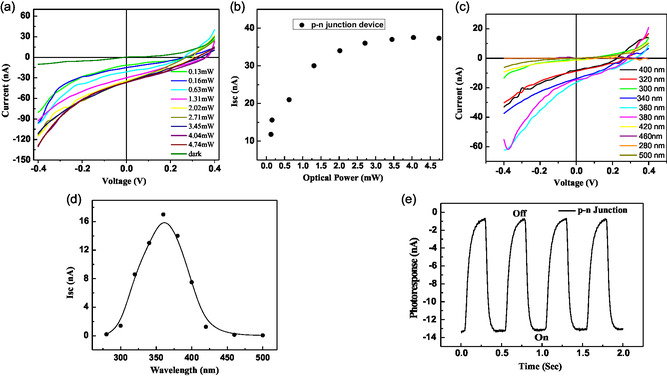
a) *I*–*V* characteristics with different optical power. b) Variation of *I*_sc_ with different optical power. c) *I*–*V* characteristics of p‐n junction device with different wavelength. d) *I*_sc_ value with different wavelengths. e) Device responses with light pulse of p‐n junction.

Next, we explicitly analyzed the dependence of photoresponse behavior of the p‐n homojunction photodiode on light wavelength and power density. **Figure** **4**a shows the *I*–*V* characteristics with different optical power varying from 0.13 to 4.74 mW. As the optical power increases, the photocurrent also rises, demonstrating a direct correlation between incident light intensity and the generated current. This trend indicates the sensitivity of the photocurrent to changes in optical power, reflecting the efficiency of the device in converting light energy into electrical current. The variation of *I*_SC_ with the optical power is shown in Figure 4b where initially the response varies linearly with increasing the optical power and it saturates after 2.5 mW. The photocurrent saturated at higher optical powers due to the saturation of charge carriers within the material. At a low optical power, there were sufficient available charge carriers to respond to the incident light, resulting in an increase in photocurrent. However, as the optical power increased, more and more charge carriers were excited, reaching a point where the material became fully populated with carriers. Beyond this point, further increases in optical power did not produce carriers, and the photocurrent saturated. Figure 4c shows the *I*–*V* characteristics at each wavelength from 300 to 500 nm when the irradiation power is fixed at 0.16 mW. Photocurrent showed distinct peaks at specific wavelengths, corresponding to the QD's absorption spectrum. However, at wavelengths either side of peak wavelength photocurrent decreased. Figure 4d shows the variation of *I*_SC_ with varying wavelengths of incident light. The peak *I*_SC_ was obtained at 360 nm and would be associated with the fact that direct transition at Γ point (Γ_25_→Γ_15_) occurs at 3.4 eV (*λ* = ≈365 nm). The magnitude of *I*_SC_ decreased for more than 360 nm and the device did not respond to light above 500 nm. This trend is consistent with optical absorption behavior shown in Figure S3a (see Supporting Information). In the UV wavelength region, the active layer absorbs light most efficiently, leading to a higher generation of charge carriers and thus an increase in photocurrent. With increasing the wavelength, the responsivity decreases which is obvious because of decreasing light‐absorbance of the QDs. Whereas for lower wavelength the UV light (*λ* < 300 nm) is blocked by the soda‐lime glass substrate of the device which is supported by the absorbance profile of the ITO substrate (see Figure S4, Supporting Information). Figure of merits of the three devices have been calculated as below. Photoresponsivity is defined as the photocurrent generated per unit power of the incident light on the effective area. The photoresponsivity was described using the following equation

(2) R(λ)=Iph(Pop×A)

where *R* is the photoresponsivity; *I*_ph_, *P*_op_, and *A* are the photocurrent, power density, and illuminated area of the device (about 4 mm^2^), respectively.

Since the shot noise from the dark current is the major contribution to the total noise in the photodetectors or photodiodes, the specific detectivity D* was calculated by^[^ ^51^, ^52^ ^]^

(3) $$D^{*}=RA^{\frac{1}{2}}/{\left(2eI_{d}\right)}^{\frac{1}{2}}$$

where *R* is the photoresponsivity, *A* is the illuminated area of device, and *I*_d_ is the dark current. External quantum efficiency (EQE) was calculated by using the formula^[^ ^53^ ^]^

(4) $$\text{EQE}\left(\%\right)=R(\lambda )\times \frac{hc}{\lambda \times q}$$

where *R*(*λ*) is the responsivity, *q* is the charge, *λ* is the wavelength of the irradiated light.

Responsivity, detectivity, and EQE were calculated using the values at zero bias voltage as summarized in Table S1 (Supporting Information). It was clear that the detectivity has been improved 22 times and reached to value 4.4 × 10^10^ Jones whereas EQE also increased 22 times for the p‐n homojunction device when compared to the values of p‐type only and n‐type only devices. As an advantage of using photodiode as photodetector, we detected photoresponse, that is, photocurrent at zero probing voltage. Photoresponse with periodic light pulse of light 360 nm with light intensity 0.16 mW has been shown in Figure 4e. The p‐type and n‐type only devices’ responses were significantly low, and they contain a large amount of noise which was not desirable for photodetection at zero bias voltage (see Figure S5, Supporting Information), whereas p‐n homojunction device exhibited a good photoresponse enough to detect the light pulse clearly even at zero bias voltage. The advantages of photodetection in photovoltaic mode such as 1) zero bias voltage, 2) no “dark” current, 3) linear, and 4) low noise (i.e., Johnson) make the p‐n homojunction layer a suitable candidate in precision applications. The response speed was calculated by using a single on/off cycle. The response speed was measured in two parts. When the 350 nm light illumination was turned on and off, the voltage was defined as the rising time (*τ*_r_) and falling time (*τ*_f_), respectively, at time intervals between 10 and 90% of the output of the normalized voltage. The *τ*_r_ and *τ*_f_ were determined from the graph for the three devices and summarized in Table S2 (see Supporting Information). The rise and fall times were 4 and 11.3 ms, respectively.

## 3 Conclusion

We have synthesized B‐doped (p‐type) and P‐doped (n‐type) Si QDs and made them soluble in two orthogonal solvents: ethanol and toluene by choosing proper monomolecular ligands namely n‐decane and 10‐undecanoic acid, respectively. Analysis combining XPS, ESR, and UPS indicated the electronic impurity doping in the QDs and underpin the Fermi level. Using two orthogonal solvents for p‐ and n‐type Si QDs, we fabricated the first p‐n homojunction diodes between ITO and aluminum electrodes by a simple spin‐casting method at room temperature in ambient conditions. The p‐n homojunction demonstrated typical *I*–*V* characteristics which clearly showed diode‐like rectification and it was used further as photodiode to detect light efficiently even at zero bias voltage conditions. The p‐n homojunction might be formed at the interface between uncapped surface Si atoms of B‐doped QDs and those of P‐doped QDs. Based on the quantum confinement effect, the photodiode device responded selectively only to UV light. However, the presence of long, insulating, and hydrophobic ligands is responsible for the poor efficiency of the device. The use of p‐type and n‐type Si QDs with inorganic capping ligands would dramatically improve device performance. Using p‐type and n‐type Si QDs with inorganic short ligands through the ligand exchange method can dramatically improve device performance.

## 4 Experimental Section

4.1

4.1.1

##### Reagents and Materials

Triethoxysilane (97%, TES) and 10‐undecenoic acid (>98.0%, UA) were purchased from Tokyo Chemical Industry (TCI) Co., Ltd. 1‐Decene (≥97.0%) was purchased from Sigma–Aldrich. Hydrofluoric acid (Ultrapur, 49%) was purchased from Kanto Chemical Co., Inc. Boric acid, phosphoric acid, toluene (HPLC grade), chloroform, ethanol (99.5), methanol, hydrochloric acid (1.0 mm), and Zn powder were purchased from Fujifilm Wako Pure Chemical Corp. Milli‐Q water (18.2 Ω cm) was supplied from the Sartorius water purification system (arium 611 UV). The reagents and chemicals were used as received, with the exception of 10‐undecenoic acid.

##### Preparation of Boron‐Doped Hydrogen Silsesquioxane

The schematic diagram of the reaction has been shown in Scheme S1, Supporting Information. TES (10 mL, 53.7 mmol) was added to a round‐bottom flask equipped with a magnetic stirring bar in an ice bath under Ar atmosphere using standard Schlenk techniques. In another flask, 165.85 mg of boric acid (2.685 mmol) was added to 20 mL Mill‐Q water. The measured pH of the solution was 5.6. The pH was adjusted to 3.0 by addition of 20 μL hydrochloric acid. This acidic mixture was added drop by drop to the TES solution over 3 min. The ice bath was removed at the same time as the addition was completed, and the flask was allowed to stand for another 2 h to allow the solution to come to room temperature. The resulting xerogel was filtered and washed with water several times until the pH of the product reached 7. The white solid obtained was dried in a vacuum overnight.

##### Preparation of Phosphorus‐Doped Hydrogen Silsesquioxane

TES (10 mL, 53.7 mmol) was added to a round‐bottom flask equipped with a magnetic stirring bar in an ice bath under Ar atmosphere on the same Schlenk line. The 20 mL aqueous solution of phosphoric acid (2.685 mmol) was added drop by drop to the TES solution over 3 min. The ice bath was removed at the same time as the addition was completed. The flask was then allowed to stand for another 2 h to allow the solution to come to room temperature. The product was filtered and washed with water several times until the pH of the product reached 7. The white solid obtained was dried in a vacuum overnight.

##### Preparation of Hydrogen‐Terminated Si QD

The dried powder was placed in a quartz crucible and transferred to a high‐temperature tube furnace with vacuum flanges. First, the inside of the quarts tube where the quartz crucible was placed was evacuated by the vacuum pump until 5 Pa and then refilled with 5%‐H2/95%‐Ar gas. This gas purge was repeated three times. The crucible was heated to 1100 °C in 5%‐H2/95%‐Ar atmosphere and kept for 2 h to yield brown solids as powder. 300 mg of the brown solid was ground in an agate mortar and then subjected to HF etching by stirring for 90 min in a mixture of 8 mL ethanol with 16 mL HF. After stirring, the solution was centrifuged at 15 000 rpm for 5 min at 10 °C in ethanol, acetonitrile, and dichloromethane in this order.

##### Preparation of a Colloidal Ink of Hydrophilic p‐type Si QDs

A colloidal ink of the hydrophilic p‐type Si QDs was prepared by hydrosilylation of 10‐undecenoic acid on the H‐Si QD derived from the boron‐doped silsesquioxane. Prior to the reaction, the 10‐undecanoic acid was degassed for 2 h at 70 °C and the pressure of 30 Pa. The dichloromethane solution of HF‐etched powder was transferred to a two‐neck round bottom flask connected to Schlenk line containing 15 mL of degassed 10‐undecanoic acid. Dichloromethane was completely removed by evacuation of the solution at room temperature using a vacuum pump. After that, the solution was heated in Ar atmosphere to the temperature of 175 °C within 3 min and kept for 3 h. Removing the mantle heater, the temperature of the solution gradually dropped to room temperature. The solution was split into four 30 mL centrifuge tubes and the rest of the tubes were filled with ethanol. Centrifugation was done at 15 000 rpm for 5 min at 10 °C. Precipitated product was dispersed in 4 mL of ethanol and hexane as an antisolvent. The centrifugation for washing the QDs was repeated at least five times. The washed QDs were dispersed in ethanol at a concentration of 10 mg mL^−1^.

##### Preparation of a Colloidal Ink of Hydrophobic n‐type Si QDs

A colloidal ink of the hydrophobic n‐type Si QDs was prepared by hydrosilylation of 1‐deceneon the H‐Si QD derived from the phosphorus‐doped silsesquioxane. The dichloromethane solution of HF‐etched powder was transferred to a two‐neck round bottom flask connected to Schlenk line containing 15 mL of degassed 1‐decene. The solution was refluxed for the reaction time as short as 10 min as the solution became transparent brown color as soon as the solution temperature reached the boiling point. By removing the mantle heater, the temperature of the solution gradually dropped to room temperature. The unreacted 1‐decene was removed by the evaporator. Afterward, the product was purified by high‐performance liquid chromatography (HPLC, Japan Analytical Industry, Japan), and the product was redispersed in toluene at a concentration of 10 mg mL^−1^.

##### Characterization of the Si QDs

XRD pattern was measured on a MiniFlex 600 (Rigaku Corp., Japan). Samples were measured at an angular step of 0.02° (time per step: 1 s per step) using CuKα (*λ* = 1.5418 Å) radiation. XPS was obtained using a Thermo Scientific Theta Probe utilizing monochromatic AlKα radiation. The samples for the XPS analysis are drop casted on the STO substrate. The XPS spectra were calibrated to the C 1*s* spectral peak a rising from adventitious hydrocarbons (284.8 eV). Optical absorption spectra were recorded using a UV‐vis spectrophotometer (JASCO V‐650, Japan). PL measurement was carried out using a modular double grating Czerny–Turner monochromator and an iHR 320 emission monochromator (1200 lines per mm of gratings) coupled to a photomultiplier tube (PMT) on a NanoLog Horiba Jovin Yvon spectrofluorometer with a 450 W xenon arc lamp. The spectral resolution of the system is around 0.3 nm. To avoid scattered excitation lights, a cut filter for 495 nm‐light was placed in front of the monochromator‐PMT setup. Electric energy levels such as Fermi level and ionization energy were estimated from the ultraviolet photoelectron spectroscopic (UPS, ThermoFisher) spectra. ESR measurement was carried out at 4.2 K using an X‐band ESR spectrometer with a magnetic ﬁeld modulation of 100 kHz to investigate the state of P donors and B donors in Si QDs. The g‐values were calibrated by means of the signals of Mn^2+^ in MgO as a standard.

##### Fabrication of p‐n Junction Photodiode

A 10 × 20 mm^2^ rectangle soda‐lime glass covered with 150 nm‐thick indium tin oxide (ITO) with a sheet resistance of 10–14 Ω sq^−1^ was used as the substrate. The ITO film was patterned by chemical etching with Zn powder and 37% HCl into a narrow strip about 2 mm wide and 20 mm long. The patterned ITO‐covered substrate was sonicated with acetone followed by isopropanol, ethanol, and then Milli‐Q water for 15 min each to remove the remaining etchant. After drying, organic contaminants on the surface were removed by exposure to VUV light (Ushio, Japan) for 30 min under a reduced pressure of 103 Pa and a N2 flow. In the Ar‐filled glove box, the ethanol ink of the p‐type Si QDs was spin coated over the ITO‐covered substrate at 1000 rpm, followed by drying at 120 °C for 30 min. Next, the toluene ink of the n‐type Si QDs was spin coated over the n‐type Si QD film on the substrate, followed by drying at 120 °C for 30 min. Finally, the cathode electrode of Al was vapor deposited. The stainless mask and the substrate were tightly adhered for patterned deposition.

##### Characterization of the Devices

All device testing was performed at room temperature under ambient conditions. Data for the current density–voltage (*I*–*V*) measurements were acquired using a Keithley 2425 source meter. A 300 W xenon lamp was used for illumination while irradiation light wavelength was varied between 280 ± 5 and 500 ± 5 nm using bandpass filters. In the *I*–*V* test, multiple devices on a substrate were measured individually and each device was characterized under light irradiation, then under dark conditions (no illumination). No obvious differences were found due to light cycling or repeated measurements on the same device within a few months of the first test. Data for the responsivity measurement was collected on a home‐built setup using illumination from the same illumination source, modulated with optical chopper (#55‐783, Edmund Optics). The anode and cathode of the device were connected to a 1 GΩ load resistor and connected to a DS‐5624A oscilloscope (Iwatsu Electric Co., Ltd) to record the modulation changes of the photovoltage under open‐circuit conditions. The frequency response of the photocurrent was displayed as a fast Fourier transform by the oscilloscope. The response time was calculated using the rise and fall times between 0.1 and 0.9, with the background of the waveform displayed on the oscilloscope as 0 and the maximum photocurrent as 1.

## Conflict of Interest

The authors declare no conflict of interest.

## Author Contributions

**Batu Ghosh**: Conceptualization (lead); Data curation (lead); Formal analysis (lead); Funding acquisition (supporting); Investigation (lead); Methodology (lead); Project administration (supporting); Resources (supporting); Software (equal); Supervision (supporting); Validation (equal); Visualization (equal); Writing—original draft (lead); Writing—review and editing (equal). **Hiroyuki Yamada**: Conceptualization (supporting); Data curation (lead); Formal analysis (lead); Funding acquisition (equal); Investigation (equal); Methodology (equal); Project administration (supporting); Resources (supporting); Software (equal); Supervision (supporting); Validation (equal); Visualization (equal); Writing—original draft (supporting); Writing—review and editing (supporting). **Kazuhiro Nemoto**: Conceptualization (supporting); Data curation (equal); Formal analysis (equal); Funding acquisition (supporting); Investigation (equal); Methodology (supporting); Project administration (supporting); Resources (supporting); Software (supporting); Supervision (supporting); Validation (equal); Visualization (supporting); Writing—original draft (supporting); Writing—review and editing (supporting). **Wipakorn Jevasuwan**: Conceptualization (supporting); Data curation (equal); Formal analysis (equal); Funding acquisition (supporting); Investigation (equal); Methodology (equal); Project administration (supporting); Resources (supporting); Software (supporting); Supervision (supporting); Validation (supporting); Visualization (supporting); Writing—original draft (supporting); Writing—review and editing (supporting). **Naoki Fukata**: Conceptualization (supporting); Data curation (equal); Formal analysis (equal); Funding acquisition (supporting); Investigation (equal); Methodology (equal); Project administration (supporting); Resources (supporting); Software (supporting); Supervision (lead); Validation (equal); Visualization (equal); Writing—original draft (lead); Writing—review and editing (equal). **Hon‐Tao Sun**: Conceptualization (supporting); Data curation (equal); Formal analysis (equal); Funding acquisition (supporting); Investigation (equal); Methodology (equal); Project administration (supporting); Resources (supporting); Software (supporting); Supervision (lead); Validation (equal); Visualization (equal); Writing—original draft (lead); Writing—review and editing (equal). **Naoto Shirahata**: Conceptualization (lead); Data curation (supporting); Formal analysis (supporting); Funding acquisition (lead); Investigation (lead); Methodology (lead); Project administration (lead); Resources (lead); Software (supporting); Supervision (lead); Validation (equal); Visualization (equal); Writing—original draft (equal); Writing—review and editing (lead).

## Supporting information

Supplementary Material

## Data Availability

The data that support the findings of this study are available from the corresponding author upon reasonable request.
