# Supplementary material for: Rational Doping Strategy to Build the First Solution‐Processed p‐n Homojunction Architecture toward Silicon Quantum Dot Photodetectors
Source: Small Sci. 2024 Oct 6;4(12):2400367. doi: 10.1002/smsc.202400367 (PMC11935281; doi:10.1002/smsc.202400367)
Supplement: Supplementary file 1 — Supplementary Material [file SMSC-4-2400367-s001.pdf]

Supporting Information

Rational Doping Strategy to Build the First Solution-Processed p-n Homojunction Architecture Towards Silicon Quantum Dot Photodetectors

Batu Ghosh,*[a,b] Hiroyuki Yamada,[a] Kazuhiro Nemoto,[a] Wipakorn Jevasuwan,[a] Naoki Fukata,*[a] Hon-Tao Sun,[a] and Naoto Shirahata*[a,c]

---

[a] Dr. B. Ghosh, Dr. H. Yamada, Dr. K. Nemoto, Dr. W. Jevasuwan, Dr. N. Fukata, Dr. H. T. Sun, Dr. N. Shirahata

Research Center for Materials Nanoarchitectonics (MANA)

National Institute for Materials Science (NIMS)

1-1 Namiki 305-0047, Japan

E-mail: batughosh@tdbcollege.ac.in, FUKATA.Naoki@nims.go.jp, SHIRAHATA.Naoto@nims.go.jp

[b] Prof. B. Ghosh

Department: Department of Physics

Institution: Triveni Devi Bhalotia College

Address: Raniganj, West Bengal, India

[c] Prof. N. Shirahata

Department: Graduate School of Chemical Sciences and Engineering

Institution: Hokkaido University

Address: Kita 13, Nishi 8, Kita-ku, Sapporo 060-8628, Japan

Table S1. Figure of merits of the p-n junction device, p-type-only device and n-type-only device.

Devices	Responsivity (μA/W)	Detectivity (Jones)	EQE (%)
p-n junction	144	4.4×10^{10}	0.0670
p-type only	6.3	2.0×10^{9}	0.0030
n-type only	6.4	2.1×10^{9}	0.0031

Table S2. Rise and fall times of the p-n junction device, p-type-only device and n-type-only device.

Devices	Rise time (msec)	Fall time (msec)
p-n junction	4.0	11.4
p-type only	6.0	15.0
n-type only	6.3	15.6

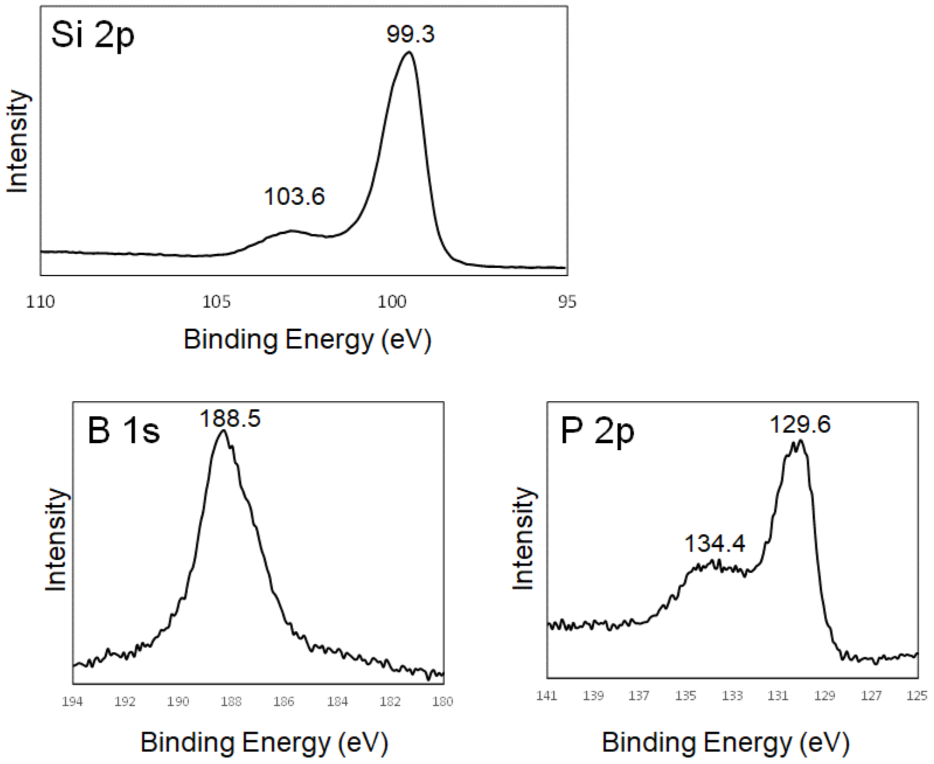

Figure S1. XPS Si2p, P2p and B1s spectra of the hydrogen-terminated Si QDs.

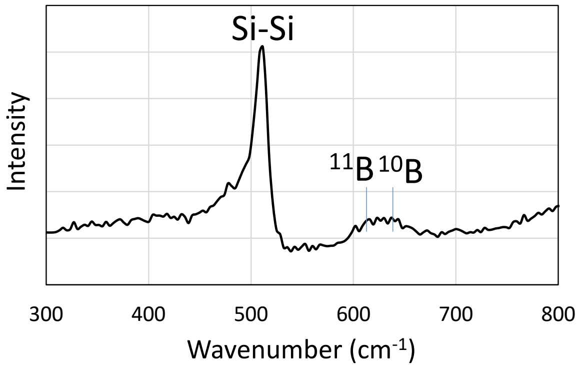

Figure S2. Raman spectrum of B-doped Si QDs terminated with hydrogen atoms.

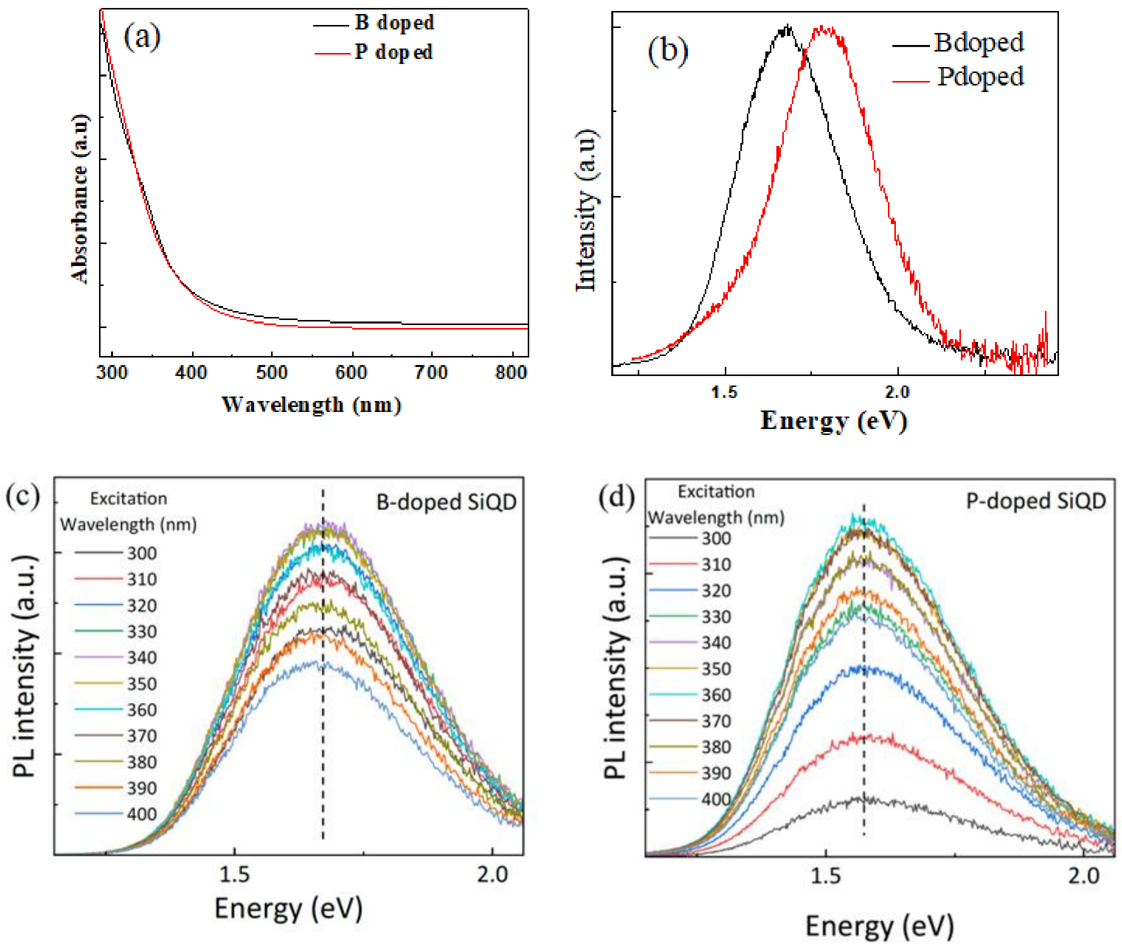

Figure S3. (a) UV-Vis and (b) PL spectra of B- and P-doped Si QDs terminated with undecanoic acid (black-color) and decane (red-color) monolayers, respectively. Excitation wavelength dependent PL spectra of (c) B- and (d) P-doped Si QDs terminated with undecanoic and decane monolayers.

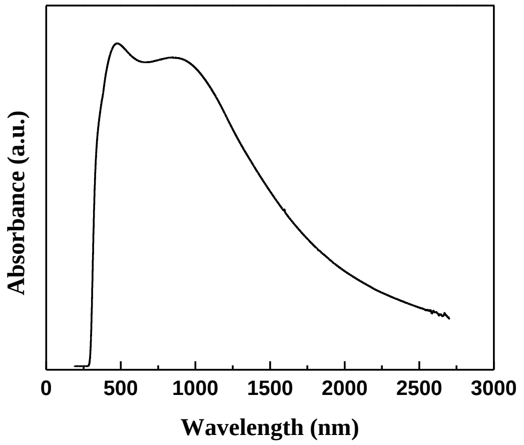

Figure S4. UV-VIS-NIR absorption spectrum of ITO-covered soda-lime glass substrate used for device fabrication.

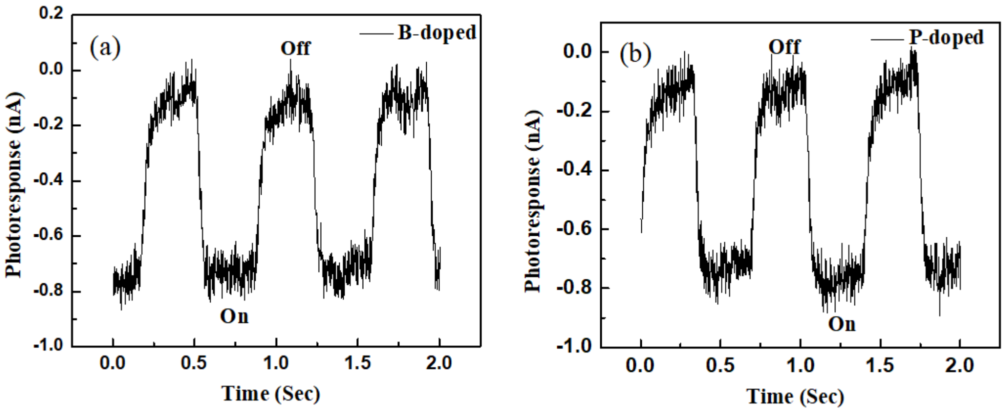

Figure S5. The typical time response curves of photocurrent for the (a) B-doped and (b) P-doped Si QD based photodiodes. The power density of the incident light (λ= 360 nm) was fixed at 0.16 mW/cm^2.
